# Post-Operative Pyoderma Gangrenosum: A Long Journey for a Patient with Myelodysplastic Syndrome

**DOI:** 10.7759/cureus.9984

**Published:** 2020-08-24

**Authors:** Ariana R Tagliaferri

**Affiliations:** 1 General Surgery, Sidney Kimmel Medical College, Thomas Jefferson University Hospital, Philadelphia, USA; 2 Internal Medicine, St. Joseph's Regional Medical Center, Paterson, USA

**Keywords:** post-operative pyoderma gangrenosum, myelodysplastic syndrome, diverticulitis, colonic resection, management, ibd

## Abstract

Pyoderma gangrenosum (PG) is an inflammatory neutrophilic dermatosis, characterized by painful and erythematous papules, pustules or vesicles that rapidly become ulcerative and necrotic. These ulcers have multiple sub-types and can develop anywhere on the body. There are different postulations as to the mechanisms of development for each sub-type. More than half of patients with PG have an underlying disease, with the highest prevalence being inflammatory bowel disease (IBD), followed by inflammatory arthritis and hematological disorders. Post-operative PG should be considered in any patient undergoing surgery who subsequently develops characteristic necrotic lesions with delayed wound healing, fever and severe localized pain. The clinical manifestations and treatment may differ slightly depending on the type and cause of PG. Herein, we present a patient with myelodysplastic syndrome and arthritis, who underwent surgery for diverticulosis complicated by colovaginal fistula formation, and subsequently developed a very prolonged course of post-operative pyoderma gangrenosum. This report will address the types of PG, their various manifestations and pathogenesis, as well as the management specific to patients with myelodysplastic syndrome. It is our intent to better understand the sub-types in order to predict and prevent post-operative PG.

## Introduction

Pyoderma Gangrenosum (PG) is an inflammatory neutrophilic dermatosis, that is neither an infectious nor gangrenous condition [1]. It is characterized by painful and erythematous papules, pustules or vesicles that rapidly progress to ulcerations with a violaceous and necrotic border [1]. Typically seen in female patients, there are around 3-10 million cases of PG per year [2,3]. Approximately 50% of those patients have underlying diseases, such as inflammatory bowel disease (IBD), inflammatory arthritis, and hematological disorders or malignancy. Of those with underlying disease, the majority tend to have IBD, although data is conflicting as to whether Crohn’s or ulcerative colitis predominates. Almost 30% of PG cases are idiopathic [1].

They can occur anywhere on the body as single or multiple lesions, ranging in sizes [1]. The types of PG include pustular, bullous, vegetative, peristomal, ulcerative and extracutaneous [1,4]. Peristomal and pustular PG are both seen in IBD, in which the lesions remain in a pustular stage for months and the severity correlates to the activity of bowel disease. Hematological malignancy is most commonly associated with bullous PG, in which painful bullae appear on the upper limbs and face spreading concentrically and progressing to superficial erosions. Vegetative PG does not have violaceous borders and is associated with Bechet’s disease, rheumatoid arthritis, diabetes mellitus and hematological disorders. Lastly, ulcerative PG presents with small follicular pustules expanding into deep violaceous ulcers with extensive erythema and induration [4]. For the purpose of this paper, we will focus on ulcerative post-operative and peristomal PG.

Ulcerative PG is the most common type and is the primary manifestation of post-operative PG [1,5]. Pathergy, a phenomenon in which skin subjected to minor or major trauma will rapidly develop PG, is the underlying principle in post-operative PG [5]. Clinically, patients develop fevers and severe pain around the incision sites [2,3,5]. Other case reports have discussed post-operative PG after mastectomy, vein grafting, skin grafting, colostomies, cesarean sections, cholecystectomy, excisional basal cell carcinoma, orthopedic procedures, appendectomies and cardiothoracic procedures [6]. Although the idea of pathergy implies that lesions will occur at the site of trauma, as seen in peristomal PG, PG lesions can also simultaneously appear at distant sites such as the mouth, neck, genitalia or arms [3]. Peristomal PG, a variant of post-operative PG is known to present as multiple sterile pustules with erythematous borders specifically around colostomy sites [7]. The time from surgery to development will vary depending on the sub-type of PG: generally, post-operative PG has been observed from four days to six weeks, while peristomal PG occurs an average of 18.4+/- 7.5 months after stoma creation [5,7]. The major diagnostic criteria include a rapidly progressive ulcerative skin lesion with irregular edges, purple-blue undermined borders with necrosis, and exclusion of other diseases [2,8]. Additionally, two minor criteria must be met, including the presence of underlying systemic diseases as mentioned above, response to corticosteroids, histopathology confirming neutrophilic infiltrates with or without lymphocytic vasculitis, or presence of pathergy [2,8].

Post-operative lesions are known to become very large and refractory to local and systemic treatments, and thus early diagnosis and intervention are crucial [5]. It is imperative to screen for and recognize those with the aforementioned underlying diseases prior to surgery to avoid surgical debridement and unnecessary treatments that may worsen PG [8]. While there are no current standardized guidelines to treat all types of PG, many drugs have been known to be effective. These medications include systemic corticosteroids, intravenous immunoglobulins, cyclosporine, azathioprine, cyclophosphamide, cyclosporine, tacrolimus, mycophenolate mofetil, methotrexate, anti-tumour necrosis factor (TNF) medications (infliximab, adalimumab) and topical treatments [7]. For refractory cases, evidence suggests that negative pressure wound therapy or hyperbaric oxygen therapy may occasionally be effective [9].

## Case presentation

**Figure 1:**
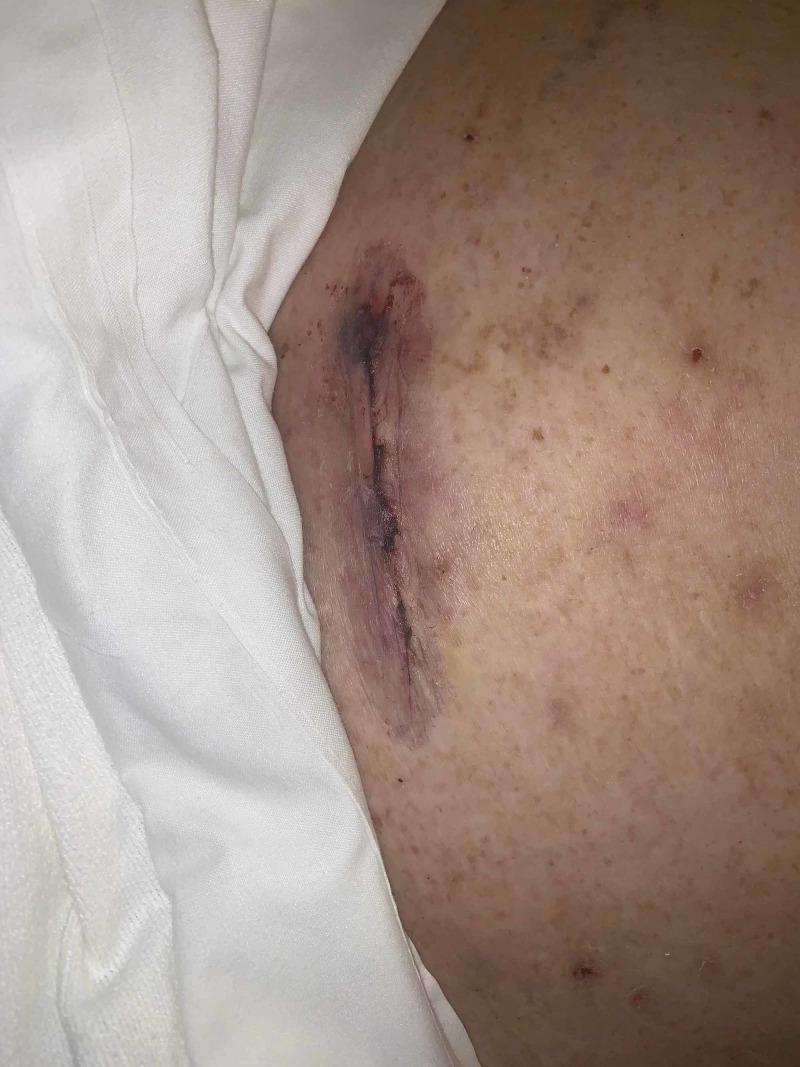
Post-operative day nine, necrotic skin and subcutaneous tissue On post-operative day nine, she developed necrotic skin and subcutaneous tissue at the port sites. The periumbilical port site was described as a rolled inflammatory gun metal grey to violaceous bordered lesion.

**Figure 2:**
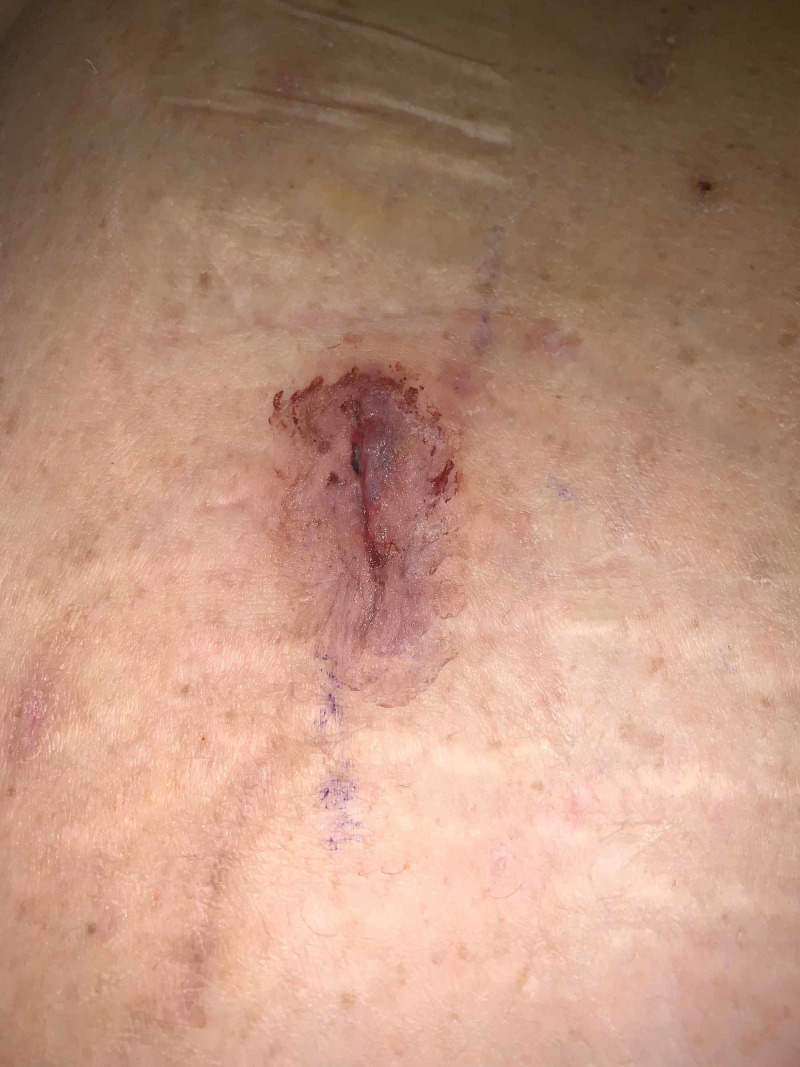
Post-operative day nine, erythematous borders of midline port site Simultaneous development of pyoderma gangrenosum lesions at midline port site.

Sixty-six-year-old female with a pertinent past medical history of inflammatory arthritis and myelodysplastic syndrome presented to the clinic with a 10-month history of recurrent diverticulitis and most recently fecal incontinence through her vagina. She was diagnosed with diverticulosis of the sigmoid colon with colo-vaginal fistula to the vagina cuff. She underwent an uncomplicated robotic sigmoid colonic resection with primary side to end 29 mm colorectal anastomosis. On post-operative day nine, she developed necrotic skin and subcutaneous tissue at the port sites, which were described as rolled inflammatory gunmetal grey to violaceous borders (Figure 1 and Figure 2).

**Figure 3:**
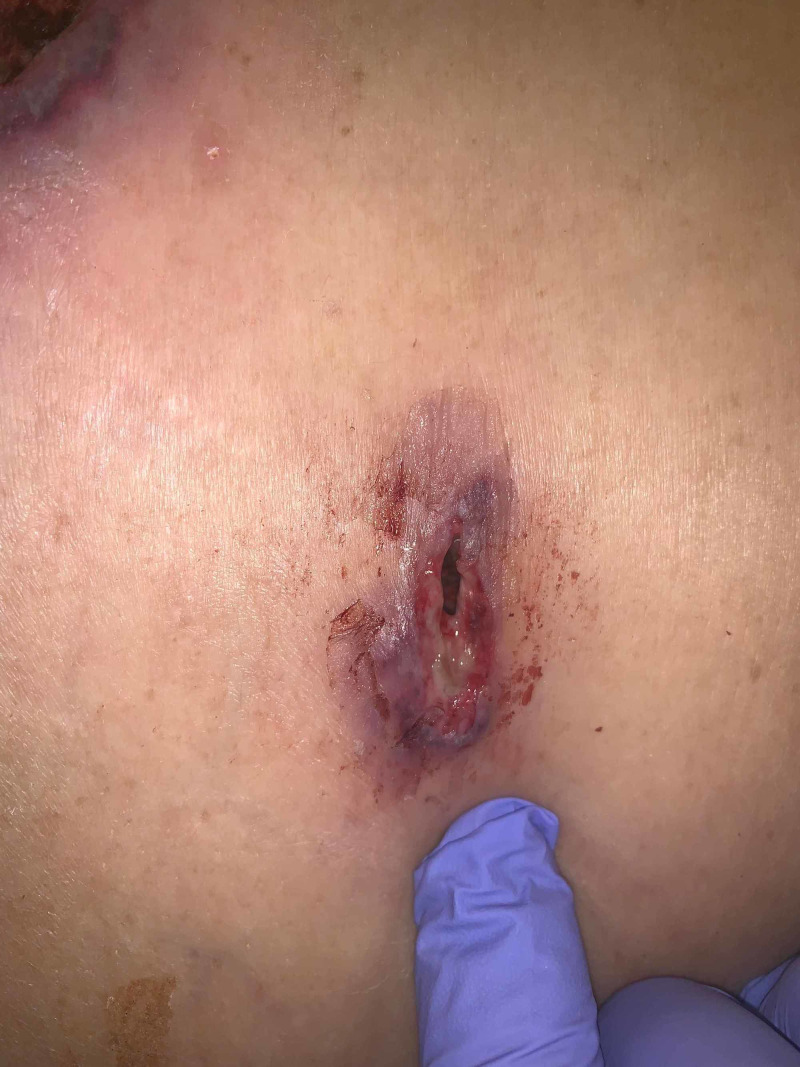
Post-operative day 10 violaceous and ulcerative borders of midline port site

She complained of lower left quadrant pain and was found to have pyrexia and leukocytosis. A gram stain and culture of the wound sites were negative, however, punch biopsies revealed PG (Figure 3).

**Figure 4:**
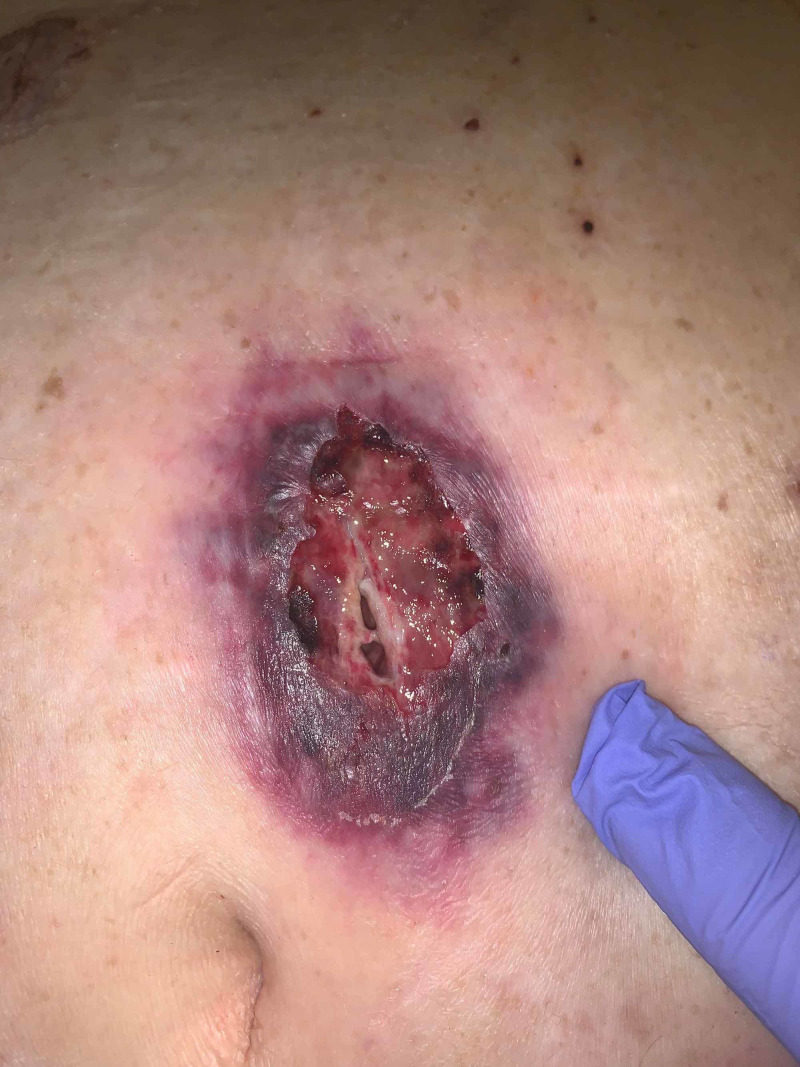
Post-operative day 11 ulcerative erosions of midline port site Wound dehiscence at initial pyoderma gangrenosum sites status post laparotomy for anastomotic leak repair.

**Figure 5:**
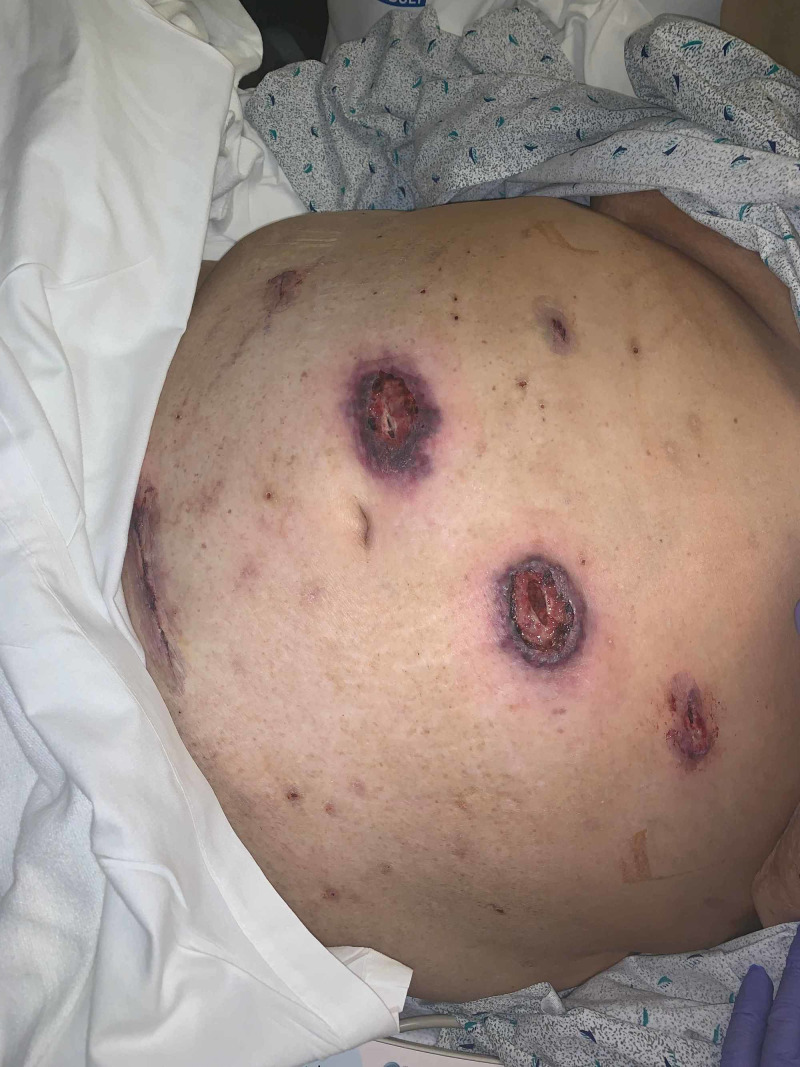
Violaceous and necrotic lesions spreading to distant sites

Prior to initiating steroid treatment, a barium enema revealed an anastomotic leak and she underwent open laparotomy for abdominal washout with ileo-ascending colon resection, ileo-ascending anastomosis, repair of the defect anastomosis with diverting loop ileostomy. Her postoperative course was further complicated by wound dehiscence and worsening areas of large violaceous ulcerations (Figure 4 and Figure 5).

**Figure 6:**
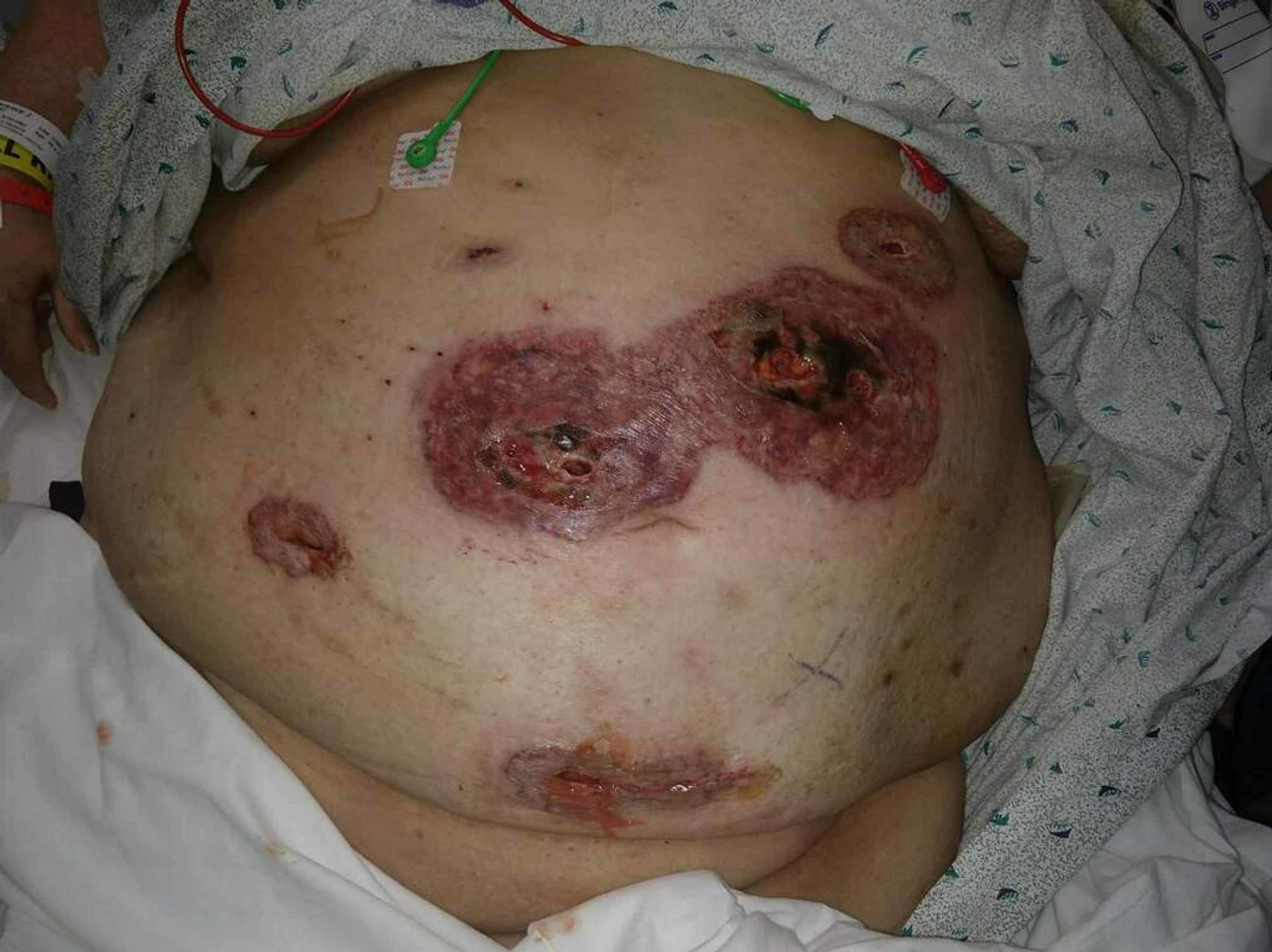
Worsening pyoderma gangrenosum lesions status post VAC therapy and steroids Post VAC therapy, medical management and wound care lesions expanded as such: 19x3x3 cm Pfannensteil wound on midline lower abdomen, 10x3x0.5 cm wound on right lower quadrant, 32x15x5 cm wound on midline abdomen extending vertically, and 7x7x4 cm sacral wound (not pictured). VAC: vacuum-assisted closure

She was treated with vacuum-assisted closure (VAC) therapy, prednisone, and infliximab. Metronidazole and clobetasol topicals were applied around the borders bi-daily. Despite treatment, her wounds continued to expand as such: 19x3x3 cm Pfannenstiel wound on midline lower abdomen, 10x3x0.5 cm wound on right lower quadrant, 32x15x5 cm wound on midline abdomen extending vertically, and 7x7x4 cm sacral wound (Figure 6).

Fifty days from her initial surgery, she was discharged to a rehabilitation center on an infliximab infusion every four weeks and a steroid taper.

Within two weeks, the patient was re-admitted with urosepsis and a new sacral wound, biopsy proven to be PG. With concerns of sepsis, steroids were initially withheld, however, a multi-disciplinary decision was to treat with ciprofloxacin, adalimumab, prednisone, VAC therapy, and total parenteral nutrition for better wound healing. This hospital course was complicated by anemia requiring transfusions, and severe protein-calorie malnutrition, starvation ketoacidosis, refeeding syndrome, and resolving toxic metabolic encephalopathy. The patient was eventually cleared for discharge two months later.

## Discussion

Evidence suggests that clinical presentations of PG are similar among different age groups: patients under 65 years old are more likely to have IBD, while patients older than 65 years are more likely to have arthritis, hematological malignancy or disorders, such as monoclonal gammopathy, myelodysplastic syndrome (MDS) or polycythemia vera [10]. A meta-analysis and systematic review illustrated that although IBD and arthritis are the most commonly associated diseases, hematological malignancies are found in up to 25% of PG patients [11]. It has been documented that 30% of all patients with a history of PG will also develop PG post-operatively [11]. Another systematic review reported that 8.6% of patients who developed post-operative PG had a hematologic disorder, 5.9% of patients had IBD, and 3.6% had rheumatoid arthritis [12].

Our patient had two underlying risk factors for the development of post-operative PG, including inflammatory arthritis and MDS. The patient also presented over the age of 65, which is consistent with the findings in the studies above. Additionally, although she did not have a diagnosis of IBD, she did undergo surgery for a colonic disease which may have increased her risk of post-operative PG. It is known that the activity of the underlying disease may reflect the severity of PG [13,14]. Thus, it can be questioned if her disease course was so prolonged due to the fact that she had multiple risk factors, as noted above.

The mechanisms underlying these variant processes are unclear, however, three ideas for which PG can develop have been postulated. The first is regarding neutrophil trafficking abnormalities, as PG ulcers express myeloperoxidase and interleukin-8 (IL-8) [4,11]. Additionally, granulocyte colony-stimulating factor (G-CSF), stimulates neutrophil differentiation and cell proliferation, and has been found to be overexpressed in patients with pre-existing PG, as well as patients undergoing chemotherapy. Although our patient was never treated with chemotherapy, this may explain the pathogenesis of PG in those with other hematological malignancies. However, the patient was taking lenalidomide, an angiogenesis inhibitor for her MDS, which should have been protective against another proposed mechanism: neutrophils generate reactive oxygen species causing overexpression of vascular endothelial growth factor (VEGF) and hypoxia inducible factor 2-alpha (HIF2a), which promote angiogenesis and perpetuation of the PG lesions. Unfortunately, the patient discontinued lenalidomide five months prior to her first resection and was subsequently not on it while hospitalized for PG. Taking lenalidomide may have decreased her risk of developing post-operative PG. Lastly, in patients with bowel disease, cross-reacting antibodies are similar to the cross-reacting antibodies found in PG ulcers [11]. This mechanism might explain why those with colon disease have higher risks of developing PG, specifically peristomal or post-operative PG. Moreover, proposes another contributing factor to the development of PG in our patient, with diverticulosis of the sigmoid colon. An increased understanding of the pathogenesis in the variants of PG may help identify therapeutic targets and a more uniform approach to treatment.

Current treatment starts with systemic corticosteroids and/or cyclosporine immune-suppression. Evidence suggests that concurrent use of intravenous methylprednisolone and immunoglobulin therapy will resolve anemia associated with PG, but can lead to progressive ulceration over the time span of 12 weeks [15]. Characteristic of ulcerative post-operative PG, our patient’s lesions developed as small follicular pustules with violaceous borders within nine days following surgery. She then developed dehiscence and ulcerations, with new lesions appearing at distant sites. During this time, she was treated with intravenous steroids, but not immunoglobulin treatment. She minimally responded to pulsed infliximab therapy, which has been shown to be effective in peristomal PG, but may lead to progressive lesions in other forms of PG [4,7]. It is clear that there is a worse response to treatment in those with multiple sites affected and in patients with hematologic disease [14,16]. These were all the case for our patient, and it may be true that the infliximab therapy worsened her condition as she had ulcerative post-operative PG rather than peristomal PG.

In steroid refractory PG, thalidomide, tacrolimus, and azathioprine and mycophenolate mofetil have been shown to be effective, however some of these were determined to be unsafe, given the patient’s history of MDS [15,17]. Researchers have searched for efficacious regimens for PG in MDS patients. The concern is treating with a drug that modulates the immune system without suppressing it. Thalidomide may have been a suitable option for the patient, as it has a similar mechanism of action to lenalidomide. Thalidomide decreases levels of TNF and interleukins. It is also an antiangiogenic medication and enhances re-epithelization by promoting migration of keratinocytes [17]. Decitabine is another medication that has shown benefit in PG patients with MDS. It is a deoxycytidine analog that at sub-therapeutic doses (0.15 mg/kg/day vs. 20-45 mg/m/day) can decrease granulation of ulcerated areas in as little as eight weeks [15]. Decitabine also reduces pain, further decreasing the need to prescribe opiates and steroids. One study showed that treatment with decitabine and prednisone completely healed an ulcer in 20 weeks [15].

## Conclusions

Pyoderma gangrenosum is a complicated disease that can present in various ways, but must be diagnosed early to prevent further complications. In post-operative PG, it is important not to debride the lesions and identify underlying diseases that put patients at risk so that one can treat PG appropriately. Patients at high risk should be educated regarding the importance of early detection and patients should be screened pre-operatively to heighten the clinical suspicion should lesions develop post-op. If surgery must be performed in patients with PG, it should be when the lesions are clinically quiescent. Treatment should be tailored to address the patient’s underlying disease, but ultimately future research should identify which pharmaceuticals will be effective given the nature of the sub-types of PG and underlying disease states.
